# Kernel Reconstruction for Delayed Neural Field Equations

**DOI:** 10.1186/s13408-018-0058-8

**Published:** 2018-02-05

**Authors:** Jehan Alswaihli, Roland Potthast, Ingo Bojak, Douglas Saddy, Axel Hutt

**Affiliations:** 1 Department of Mathematics and Statistics, University of Reading, Reading, UK; 2 Department of Mathematics, Faculty of Education, Misurata University, Misurata, Libya; 3 Division for Data Assimilation (FE12), Deutscher Wetterdienst, Offenbach, Germany; 4 Centre for Integrative Neuroscience and Neurodynamics (CINN), Department of Psychology, University of Reading, Reading, UK

**Keywords:** Neural fields, Integral equations, Fixed-point theorem, Inverse problems, Regularization

## Abstract

Understanding the neural field activity for realistic living systems is a challenging task in contemporary neuroscience. Neural fields have been studied and developed theoretically and numerically with considerable success over the past four decades. However, to make effective use of such models, we need to *identify their constituents* in practical systems. This includes the determination of model parameters and in particular the reconstruction of the underlying effective connectivity in biological tissues.

In this work, we provide an *integral equation approach* to the reconstruction of the *neural connectivity* in the case where the neural activity is governed by a delay neural field equation. As preparation, we study the solution of the direct problem based on the Banach fixed-point theorem. Then we reformulate the inverse problem into a *family of integral equations* of the *first kind*. This equation will be vector valued when several neural activity trajectories are taken as input for the inverse problem. We employ *spectral regularization techniques* for its stable solution. A *sensitivity analysis* of the regularized kernel reconstruction with respect to the input signal *u* is carried out, investigating the Fréchet differentiability of the kernel with respect to the signal. Finally, we use numerical examples to show the feasibility of the approach for kernel reconstruction, including numerical sensitivity tests, which show that the *integral equation approach* is a very stable and promising approach for practical computational neuroscience.

## Introduction

In recent years, studying the activity of neural tissue and development of mathematical and numerical techniques to understand neural processes has led to improved neural field models. Since the early work of Wilson, Cowan and Amari in the 1970s neural field models have become an effective tool in neuroscience [1–4].

The neural networks occurring in nature are typically complex systems sporting a large variety of properties in space and time. Simplifying their analysis is generally difficult—in particular when one considers the many billions of neurons of the entire human nervous system, where each of these neurons can be considered as a complex biological system by and in itself, cf. [5]. However, neural field models describe these complicated system mathematically in a few equations, essentially by using the large number of neurons to achieve simplification in terms of mass action. Thus these models consider averages of the neural activity as a dynamical variable, and averages of neural properties as parameters. The derivation of neural models from properties of single neurons and their networks, and the analysis of the resulting activity, remains a major focus of current research [1, 6–11].

In this century, there are many papers on the neural field equation with and without delays. Some of the studies provide a framework for the existence, uniqueness and stability of the solutions of the neural field equation such as [8–15], while others consider building effective methods to investigate and assimilate the neural field activities, see for example [16–21] with techniques of Data assimilation and Inverse Problems applied to the case without delays. Recently, Nogaret et al. [7] built a model construction method using an optimization technique to assimilate neural data to determine parameters in a detailed neural model including delay.

A challenge often encountered in the study of living systems is to estimate a *spatial connectivity kernel* *w*. In a neural system this connectivity kernel usually corresponds to the synaptic footprint, i.e., the connections from a neuron to others by synapses forming between its branching axon and their dendritic trees. Typically, measurements are available for the activity function *u* at particular spatial locations, e.g., where neurons are patch- clamped or electrodes are placed in the extracellular medium. The task then becomes to derive the spatial connectivity from these experimental data. This approach limits the estimation of connectivity to the set of spatial locations of measurements. In the present work, we propose to improve this conventional approach by studying the inverse problem where the *full activity function* *u* is given at each location in a given spatial domain and the underlying spatial connectivity is derived. The problem of having limited measurements is part of subsequent work combining inverse techniques with state estimation techniques. Here, we focus on the problem to reconstruct the kernel *w* when *u* is known.

The present work considers neural field models that involve delayed spatial interactions and where the delay may depend on the distance between spatial locations [11, 14, 22]. We will assume that the delay function $D(r,r')$ between spatial locations *r*, $r'$ is known. For instance, this is the case when the delay is linked to the *geometry* of the problem, e.g., when $D(r,r')\sim \Vert r-r' \Vert $, the distance between the points *r* and $r'$ in some domain *Ω*. This assumption is common in practice, since for direct neural connections the delay is essentially the distance divided by the signal propagation speed, which can be assumed to be a universal constant in a first approximation.

Neural field models consider spatially nonlocal interactions, which may be expressed equivalently either by higher orders of spatial derivatives or by spatial integrals [22, 23]. In the first part of this paper, we will show how the methods used in [12] can be modified to study existence and stability of solutions in a neural field model with delay. The basic idea is to split the integral operators under consideration into parts with positive and negative temporal arguments. As a result we obtain a direct and flexible basic existence proof for a delay neural field equation, which includes a constructive method based on integral equations only. These results have been derived by other authors [8, 10, 11, 24] with more sophisticated techniques, but it is non-trivial that the arguments used for neural fields without delay are applicable to the delay case, and the approach in our Sect. 2, based on several relatively simple functional analytic arguments, is of interest by itself.

Second, we will show that the kernel reconstruction problem for the delay neural field equation can be reformulated into a *family of integral equations* of the *first kind*. When several trajectories of neural activity are given, the family of integral equations is vector valued. This turns out to be an ill-posed problem, for smooth neural activity it is even exponentially ill posed. To formulate stable numerical methods for its solution, we need to employ regularization. Here, we use a spectral approach to classical Tikhonov regularization [25–27]. We then study the *sensitivity* of the mapping $u \mapsto w$ showing that its regularized version is Fréchet differentiable, and we calculate the derivative by means of integral equations.

In the third part of the paper, we show by a numerical study that the kernel reconstruction from a delay neural field is feasible. We numerically solve the family of integral equations under consideration by a collocation method and provide a study of reconstructions based on the regularization of the ill-posed integral operators under consideration. This includes a study of the influence of measurement noise on the reconstruction quality and tests of the role of the regularization parameter.

We start with a concise version of the equations in Sect. 2, and in Sect. 3 prepare our inverse approach by a study of the existence for the delay neural field equation. The central section, Sect. 4, serves to develop a family of integral equations to solve the inverse problem for the delay neural field equation. The numerical realization of the approach is shown in Sect. 5, where we demonstrate that with an appropriate regularization the inverse problem is solvable, i.e., prescribed kernels can be constructed and reconstructed kernels generate a neural environment leading to the prescribed neural behaviour.

## The Mathematical Model

In neural dynamics, neurons send electrical spikes to each other through axons terminating in synapses. Let $u(r_{j},t)$ denotes the average membrane potential of the *j*th neuron located at position $r_{j}$ at time *t* in a network of *N* units. Let $W(r_{j},r_{i})$ be the average connectivity strength between neuron at position $r_{i}$ and neuron at position $r_{j}$. The function *f* is the activation rate or firing rate function, which describes the conversion of the membrane potential $u(r_{j},t)$ into a spike train $S(r_{i},t)=f[u(r_{i},t)]$, which is then leading to an excitation of neurons at location $r_{j}$ with strength $W(r_{j},r_{i}) S(r_{i},t)$. The dynamics of the excitation is now described by the ODEs

1 $$ \tau\frac{du}{dt}(r_{j},t) = -u(r_{j},t) + \sum_{i=1}^{N} W(r_{j},r_{i}) f \bigl[u(r_{i},t) \bigr]. $$

This combination of an exponential decay with characteristic time *τ* and a sum of excitation terms is commonly called a ‘leaky integrator model’. The sum represents the net-input to unit *j*, i.e., the weighted sum of activity delivered by all units *i* that are connected to unit *j* with a connection strength $W(r_{j},r_{i})$; cf. [12, 28]. The continuous version of (1) is obtained by considering neurons which are continuously distributed over the space *Ω*, e.g., in a plane with $\varOmega\in \mathbb {R}^{2}$ or $\varOmega\in \mathbb {R}^{3}$ and by replacing the sum by an integral. This leads to the simplest form of the Amari neural field equation [4],

2 $$ \tau\frac{\partial u}{\partial t}(r,t) = -u(r,t) + \int_{\varOmega}w\bigl(r,r'\bigr) f \bigl[u \bigl(r',t\bigr) \bigr] \, dr', \quad r \in\varOmega. $$

Here $u(r,t)$ indicates a neural field representing the activity of the population of neurons at position *r* and time *t*. The second term on the right-hand side represents the synaptic input, where *f* is the activation (or firing rate) function of a single neuron. The kernel $w(r,r')$ is often referred to as the *synaptic footprint* [29–31] or the *connectivity* function [12, 14, 32, 33]. It presents the strength of the connection between neurons located at *r* and $r'$. The function *w* incorporates three different kinds of meaning: the existence of a connection in the first place, if $w \neq0$, the functional effect of either excitation, if $w > 0$, or inhibition, if $w < 0$, and finally the strength of the connectivity via $|w|$ [4, 12, 34].

Although the neural field equation (2) represents several biological mechanisms, this form still neglects any *delay* between spatial locations. In reality, finite transmission speeds in axons, synapses and dendrites cause a functionally significant delay. Taking it into account, the neural field equation involving delayed interactions becomes

3 $$ \tau\frac{\partial u}{\partial t}(r,t) = -u(r,t) + \int_{\varOmega}w\bigl(r,r'\bigr) f \bigl[ u \bigl(r',t - D\bigl(r,r'\bigr) \bigr) \bigr] \,dr',\quad r \in\varOmega, $$

where the delay is typically assumed to be $D(r,r')\simeq\tilde{D}(r,r')/v$, i.e., the total length of the neural fibers *D̃* connecting locations *r* and $r'$, divided by *v*, the finite transmission speed of neural signals (action/post-synaptic potentials) along those fibers. In general, *D* is not constant but continuous. Equation (3) is accompanied by initial conditions. These depend on the geometry of the spatial domain and the specific temporal dynamics under study. They are considered in detail in the subsequent sections.

The existence of solutions to the neural field equation (3) has been investigated in various papers already [10–12]. For example, Potthast and beim Graben [12] provide the proof of existence and its analysis in the case of no delay, i.e. for $D(r,r')=0$. In addition, Faugeras and Faye [10], in their Theorem 3.2.1, state the general existence of solutions with a reference to the generic theory of delay equations, based on work such as [35]. We also point out the work of Van Gils et al. [8] employing the sun–star calculus for their analysis and [24] in which the local bifurcation theory for delayed neural fields was developed. Here, we develop arguments on how to use the basic functional analytic calculus to work for the delay case as well, with the goal to present a short and elementary approach which is easily accessible.

## The Delay Neural Field Equation

In this work, we study the neural field equation (3) on some bounded domain $\varOmega\subset \mathbb {R}^{m}$ in a space with dimension $m =2$ or $m=3$. We assume that the transmission delay $D(r,r')$ of neural excitation or inhibition between $r'$ and *r* is bounded on $\varOmega\times\varOmega$, i.e. there is a constant $c_{T}$ such that

4 $$ \bigl\vert D\bigl(r,r'\bigr) \bigr\vert \leq c_{T}, \quad r,r' \in\varOmega. $$

At time $t\in \mathbb {R}$, the neural fields $u(r,t)$ at a point $r \in\varOmega $ might receive excitations from the past with a maximal delay of $c_{T}$. Working on the time interval $[0,\rho]$ with $\rho>0$, equation (3) is complemented by initial conditions in the time interval $[-c_{T},0]$. The *initial condition* for the delay neural field equation is given by

5 $$ u(r,t)= u_{0} (r, t), \quad(r,t) \in\varOmega\times[-c_{T},0]. $$

We lay ground for our inverse and sensitivity analysis by a basic derivation of the unique solvability of equation (3), using tools from functional analysis and integral equations. Our investigation here makes a smoothness assumption for the activity function *f* and the connectivity kernel *w*. We consider a continuous activation function $f(s)$ for $s\in\mathbb{R}$ and an activation threshold *η*. This function may be interpreted as the mass action probability of neurons firing if their membrane potential is over the threshold, and can be derived from a stochastic neuron models [6, 36]. Typically [1, 29], *f* is approximated by the logistic sigmoidal function

6 $$ f(s)=\frac{1}{1+e^{- \sigma(s-\eta)}}, \quad s \in \mathbb {R}, $$

with some steepness parameter $\sigma>0$ and threshold *η*. For the function $f: \mathbb {R}\rightarrow \mathbb {R}^{+}$ we note that

7 $$ f(s) \subset[0,1], \quad s\in \mathbb {R}. $$

Here, we will work with general Lipschitz continuous functions *f* satisfying this condition. We assume that the kernel *w* satisfies

- (H1) $w(r,\cdot)\in L^{1}(\varOmega)$, $\forall r \in\varOmega\subset \mathbb{R}^{m}$,

such that we obtain a *well-defined* integral of the form

$$g(r,s) := \int_{\varOmega} w\bigl(r,r'\bigr) f\bigl(u \bigl(r',s-D\bigl(r,r'\bigr)\bigr)\bigr) \,dr', \quad r \in\varOmega, s \in \mathbb {R}. $$

The condition

- (H2) $\sup_{r \in\varOmega} \|w(r,\cdot)\|_{L^{1}(\varOmega)} \leq C_{1} $,

with some constant $C_{1}$ leads to *g* being *bounded* on $\varOmega \times \mathbb {R}$. We need $g(r,s)$ to be *continuous* in dependence of *r* and *s*, which for continuous functions *u* and *D* is achieved by the additional condition

- (H3) $\|w(r,\cdot)-w(r^{\ast},\cdot)\|_{L^{1}(\varOmega)} \rightarrow0$ for $|r-r^{\ast}| \rightarrow0$.

Now, existence is given by the following result.

### Theorem 3.1

(Existence)

*If the kernel* *w* *satisfies* (H1)*–*(H3), *and if the delay term* *D* *is bounded continuous*, *i*.*e*., *if we have* $D \in\operatorname{BC}(\varOmega\times\varOmega,\mathbb{R}^{+})$, *then for any* $T>0$ *and for any initial field* $u_{0}$ *as given by the initial condition* (5) *there exists a unique solution* $u \in C^{1} (\varOmega\times[0,T])$ *to the delay neural field* (3) *on* $[0,T]$.

### Proof

We first need some preparations. We will need to split the function $u(r,s-D(r,r'))$ into the part where the time variable $t=s-D(r,r')$ is in $(0,T]$ and where $t=s-D(r,r')$ is in $[-c_{T},0]$. This is carried out by defining

8 $$ \chi_{+}(r,t) := \textstyle\begin{cases} 1, & t > 0, \\ 0, & t \leq0, \end{cases} $$

and $\chi_{-}(r,t) := 1 - \chi_{+}(r,t)$. The function $\chi_{-}$ is equal to 1 for negative time arguments and we have $1 = \chi_{+}+\chi_{-}$. For studying the existence of solutions of the delay neural field equation (3) we define the operators

9 $$ (A_{1}u) (r,t) := \int_{0}^{t} -\frac{u(x,s)}{\tau} \,ds, \quad r \in \varOmega\mbox{ and } t \leq0, $$

and

10 $$\begin{aligned} \bigl(A^{\pm}_{2}u\bigr) (r,t) :=& \frac{1}{\tau} \int_{0}^{t} \int_{\varOmega}w\bigl(r,r'\bigr) \chi_{\pm} \bigl(r,s-D\bigl(r,r'\bigr) \bigr) \\ &{}\cdot f \bigl[u \bigl(r',s-D \bigl(r,r'\bigr) \bigr) \bigr] \,dr' \,ds \end{aligned}$$

for $r \in\varOmega$ and $t \in[0,T]$. By integration with respect to time the solution of (3) can be reformulated as

11 $$\begin{aligned} &u(r,t)-u(r,0) \\ &\quad = \frac{1}{\tau} \int_{0}^{t} \biggl\{ -u(r,s) + \int_{\varOmega}w\bigl(r,r'\bigr) f \bigl[ u \bigl(r',s-D\bigl(r,r'\bigr) \bigr) \bigr] \,dr' \biggr\} \,ds \end{aligned}$$

for $r\in\varOmega$ and $t \in[0,\rho]$ with an auxiliary parameter *ρ*. Differentiating equation (11) with respect to time, we return to the delay neural field equation (3). We can now split the operators as follows:

12 $$\begin{aligned} &\frac{1}{\tau} \int_{0}^{t} \int_{\varOmega}w\bigl(r,r'\bigr) f \bigl[ u \bigl(r',s-D\bigl(r,r'\bigr) \bigr) \bigr] \,dr' \,ds \\ &\quad = \bigl(A^{+}_{2}u\bigr) (r,s) + \bigl(A^{-}_{2}u\bigr) (r,s) \\ &\quad = \bigl(A^{+}_{2}u\bigr) (r,s) + \bigl(A^{-}_{2}u_{0} \bigr) (r,s), \end{aligned}$$

where the last equality is obtained from

$$\begin{aligned} \tau\bigl(A^{-}_{2}u\bigr) (r,t) = & \int_{0}^{t} \int_{\varOmega}w\bigl(r,r'\bigr) \chi_{-} \bigl(r,s-D\bigl(r,r'\bigr)\bigr)f \bigl[ u \bigl(r',s-D \bigl(r,r'\bigr) \bigr) \bigr] \,dr' \,ds \\ = & \int_{\varOmega}\int_{0}^{t} w\bigl(r,r'\bigr) \chi_{-}\bigl(r,s-D\bigl(r,r'\bigr)\bigr)f \bigl[ u \bigl(r',s-D\bigl(r,r'\bigr) \bigr) \bigr] \,ds \,dr' \\ = & \int_{\varOmega}\int_{0}^{D(r,r')} w\bigl(r,r'\bigr)f \bigl[ u \bigl(r',s-D\bigl(r,r'\bigr) \bigr) \bigr] \,ds \,dr' \\ = & \int_{\varOmega}\int_{0}^{D(r,r')} w\bigl(r,r'\bigr)f \bigl[ u_{0} \bigl(r',s-D\bigl(r,r'\bigr) \bigr) \bigr] \,ds \,dr' \\ = & \int_{\varOmega}\int_{0}^{t} w\bigl(r,r'\bigr) \chi_{-}\bigl(r,s-D\bigl(r,r'\bigr)\bigr)f \bigl[ u_{0} \bigl(r',s-D\bigl(r,r'\bigr) \bigr) \bigr] \,ds \,dr' \\ = & \int_{0}^{t} \int_{\varOmega}w\bigl(r,r'\bigr)\chi_{-} \bigl(r,s-D\bigl(r,r'\bigr)\bigr)f \bigl[ u_{0} \bigl(r',s-D\bigl(r,r'\bigr) \bigr) \bigr] \,dr' \,ds \\ = & \tau\bigl(A^{-}_{2}u_{0}\bigr) (r,t) \end{aligned}$$

using $u(r,t)=u_{0}(r,t)$ for $t\leq0$. With $A:=A_{1}+A^{+}_{2}$ the delay neural field equation is equivalent to the *fixed-point equation*

13 $$ u(r,t)=u(r,0)+ \bigl(A^{-}_{2}u_{0} \bigr) (r,t) + (Au) (r,t),\quad r \in\varOmega\mbox{ and } t \in[0, \rho]. $$

Here, the function $u(r,t)$ needs to be considered on $\varOmega\times [0,\rho]$ only and we can study the fixed-point equation in $\operatorname{BC}(\varOmega \times[0,\rho ])$. Any solution to equation (13) will be continuously differentiable with respect to time and satisfy the delay neural field equation (3). We now show that for sufficiently small parameter $\rho>0$ the operator *A* is a contraction on the space $\operatorname{BC}(\varOmega \times[0,\rho ])$ equipped with its canonical norm

14 $$ \Vert v \Vert _{\rho} := \sup_{r\in\varOmega, t\in[0,\rho]} \bigl\vert v(r,t) \bigr\vert . $$

We will carry out these arguments in four steps, I–IV.

I. For the linear operator $A_{1}$ given by equation (9), we follow [12], Lemma 2.5, and estimate

15 $$ \bigl\Vert (A_{1}u) \bigr\Vert _{\rho}= \sup _{r\in\varOmega, t\in[0,\rho ]} \bigl\vert (A_{1}u) (r,t) \bigr\vert \leq \frac{\rho}{\tau}\sup_{r\in\varOmega, t\in[0,\rho]} \bigl\vert u(r,t) \bigr\vert = \frac{\rho}{\tau} \Vert u \Vert _{\rho}, $$

i.e., the operator $A_{1}$ maps the space $\operatorname{BC}(\varOmega \times[0,\rho])$ boundedly into itself and by equation (15) the operator norm is bounded by $\rho/\tau$.

II. We define

16 $$ J u(r,t) := \frac{1}{\tau} \int_{\varOmega} w\bigl(r,r'\bigr) \chi_{+} \bigl(r,t-D\bigl(r,r'\bigr)\bigr) f \bigl( u\bigl(r',t-D \bigl(r,r'\bigr)\bigr) \bigr) \,dr', $$

for $x \in\varOmega$ and $t \geq0$, and follow [12], Lemma 2.5, to estimate

17 $$\begin{aligned} &\bigl\vert J u_{1}(r,t)-J u_{2}(r,t) \bigr\vert \\ &\quad \leq \frac{1}{\tau} \int_{\varOmega}\bigl\vert w\bigl(r,r'\bigr) \bigr\vert \chi_{+}\bigl(r,t-D\bigl(r,r'\bigr)\bigr) \\ &\qquad {} \cdot\bigl\vert f \bigl[u_{1}\bigl(r',t-D \bigl(r,r'\bigr)\bigr) \bigr] - f \bigl(u_{2} \bigl(r',t-D\bigl(r,r'\bigr)\bigr) \bigr) \bigr\vert \,dr' \end{aligned}$$

for $x \in\varOmega$ and $t \in[0,\rho]$. First, using the Lipschitz continuity of the function *f* with Lipschitz constant $L>0$, using $C_{1}$ given in (H2) we obtain

18 $$\begin{aligned} & \bigl\vert J u_{1}(r,t)-J u_{2}(r,t) \bigr\vert \\ &\quad\leq\frac{L}{\tau} \int_{\varOmega}\bigl\vert w\bigl(r,r'\bigr) \bigr\vert \chi_{+}\bigl(r,t-D\bigl(r,r'\bigr)\bigr) \\ &\qquad {}\cdot \bigl\vert u_{1}\bigl(r',t-D\bigl(r,r'\bigr) \bigr) - u_{2}\bigl(r',t-D\bigl(r,r'\bigr) \bigr) \bigr\vert \,dr' \\ &\quad\leq\frac{L C_{1}}{\tau} \sup_{r' \in\varOmega} \bigl\{ \chi _{+}\bigl(r,t-D\bigl(r,r'\bigr)\bigr) \bigl\vert u_{1}\bigl(r',t-D\bigl(r,r'\bigr)\bigr)- u_{2}\bigl(r',t-D\bigl(r,r'\bigr)\bigr) \bigr\vert \bigr\} \\ &\quad\leq\frac{L C_{1}}{\tau} \| u_{1}-u_{2} \|_{\varOmega\times[0,\rho]} \end{aligned}$$

for $r \in\varOmega$ and $t \in[0,\rho]$.

III. Integration of equation (18) with respect to $t \in [0,\rho]$ leads to

19 $$ \bigl\Vert A_{2}^{+}(u_{1}) - A_{2}^{+}(u_{2}) \bigr\Vert _{\rho} \leq\frac{\rho L C_{1}}{\tau} \| u_{1}-u_{2} \|_{\rho}, $$

where $\Vert \cdot \Vert _{\rho}$ as defined in equation (14). Now, for the operator *A* we obtain the estimate

20 $$\begin{aligned} \bigl\Vert A(u_{1})-A(u_{2}) \bigr\Vert _{\rho} =& \bigl\Vert A_{1}(u_{1} - u_{2}) + A_{2}^{+}(u_{1}) - A_{2}^{+}(u_{2}) \bigr\Vert _{\rho} \\ \leq&\frac{\rho}{\tau} \Vert u_{1}-u_{2} \Vert _{\rho}+\frac{\rho L C_{1}}{\tau} \Vert u_{1}-u_{2} \Vert _{\rho} \\ \leq& q \Vert u_{1}-u_{2} \Vert _{\rho}, \end{aligned}$$

with

21 $$ q:=\frac{\rho}{\tau}(1+LC_{1}) . $$

In the case where *ρ* is small enough to guarantee that $q<1$ by equation (20), we have shown that *A* is a contraction on $\operatorname{BC}(\varOmega\times[0,\rho], \Vert \cdot \Vert _{\rho})$.

IV. According to the Banach fixed-point theorem, there is one and only one fixed point $u^{\ast}$ for the fixed-point equation (13). We have shown the existence of a unique solution $u(x,t)$ for all $t \in[0,\rho]$. Now, the same argument applied to the interval $[\rho,2\rho]$ and subsequent intervals $[2\rho,3\rho]$ etc. in the same way. This leads to the existence and uniqueness result on the interval $[0,T]$. □

### Remark

We note that the proof also works when some bounded continuous forcing term $I(r,t)$, $r\in\varOmega$, $t \in[0,T]$, is added to the neural field equation (3). It leads to an additional term in Eq. (13), for which all arguments remain valid.

It is well known [21, 27] that Banach’s theorem also provides a constructive method to calculate the fixed point by successive iterations. Let $u_{1}$ be a starting function. Then the sequence defined by

22 $$ u_{n+1} := u_{0} + A^{-}_{2}(u_{0}) + A(u_{n}),\quad n = 1,2,3,\ldots, $$

converges to the unique fixed point $u^{\ast}$. An error estimate for this iteration process based on equation (20) is obtained from

23 $$\begin{aligned} \bigl\Vert u_{n+1} - u^{\ast} \bigr\Vert = & \bigl\Vert u_{0} + A^{-}_{2}(u_{0}) + A(u_{n}) - \bigl(u_{0} + A^{-}_{2}(u_{0}) + A\bigl(u^{\ast}\bigr)\bigr) \bigr\Vert \\ = & \bigl\Vert A(u_{n}) - A\bigl(u^{\ast}\bigr) \bigr\Vert \\ \leq& q \bigl\Vert u_{n} - u^{\ast} \bigr\Vert . \end{aligned}$$

Induction immediately leads to the full error estimate

24 $$ \bigl\Vert u_{n+1} - u^{*} \bigr\Vert \leq q^{n} \bigl\Vert u_{1} - u^{*} \bigr\Vert , \quad n\in \mathbb{N}. $$

For our numerical calculations we have, however, instead used Runge–Kutta or Euler methods applied to the differential form of the delay neural field equation.

## The Inverse Problem of Kernel Reconstruction with Delays

We now come to the *kernel reconstruction* from given dynamical neural patterns with delay. We first formulate a *regularized kernel reconstruction* approach based on *integral equations* in Sect. 4.1, then we carry out a *sensitivity analysis* in Sect. 4.2.

### Kernel Reconstruction with Delays

Usually, we will observe the dynamical evolution of some pattern for a system under consideration. More generally, observations may start from different inital patterns that lead to different dynamical trajectories in the phase space. If we have *N* such trajectories, the task is to find the kernel which will predict these trajectories when the *N* initial conditions are provided. In more detail, the goal of this section is to investigate the *inverse problem* of *kernel reconstruction* for the delay neural field equation (3). We assume that

- the nonlinear activation function $f: \mathbb {R}\rightarrow \mathbb {R}^{+}$ is known, and
- the delay function $D: \varOmega\times\varOmega\rightarrow[0,c_{T}]$ is given.

The task is to find a kernel $w(r,r')$ for $(r,r') \in\varOmega$ given the time-dependent neural activation patterns $u^{(\xi)}(r,t)$ for $(r,t)\in\varOmega\times[0,T]$ corresponding to initial conditions $u_{0}^{(\xi)}(r,t)$ for $(r,t) \in\varOmega\times[-c_{T},0]$ according to equation (5), where $\xi=1,\ldots,N$.

Here, we reformulate the inverse problem into a *family of integral equations of the first kind* and study their solution by regularization methods. As a first step, we define

25 $$ \phi^{(\xi)}(r,s):= f \bigl[ u^{(\xi)} (r,s ) \bigr],\quad(r,s) \in\varOmega\times[-c_{T},T ] , $$

and

26 $$ \psi^{(\xi)}(r,t):= \tau\frac{\partial u^{(\xi )}}{\partial t}(r,t) + u^{(\xi)}(r,t),\quad(r,t) \in\varOmega\times[0,T], $$

for $\xi=1,2,\ldots,N$. With the integral operator *W* defined by

27 $$ (W\phi) (r,t):= \int_{\varOmega}w\bigl(r,r'\bigr) \phi \bigl(r',t-D\bigl(r,r'\bigr) \bigr) \,dr' , \quad(r,t) \in\varOmega\times[0,T], $$

the inverse problem is reformulated as the equation

28 $$ \psi^{(\xi)}(r,t)=\bigl(W\phi^{(\xi)}\bigr) (r,t), \quad(r,t) \in\varOmega\times[0,T ], $$

with $\xi=1,2,\ldots,N$, where the kernel $w(r,r')$ with $r,r' \in\varOmega$ of the linear operator *W* is unknown. Equation (28) can be written as

29 $$ \psi=W\phi, $$

with $\phi=(\phi^{(1)},\phi^{(2)},\ldots,\phi^{(N)})^{T}$ and $\psi=(\psi^{(1)},\psi^{(2)},\ldots,\psi^{(N)})^{T}$, where we search for the unknown operator *W*. An alternative is to rewrite equation (28) as

30 $$ \psi_{r}(t)= \int_{\varOmega}\phi\bigl(r',t-D\bigl(r,r' \bigr)\bigr) w_{r}\bigl(r'\bigr) \,dr', \quad t \in[0,T] , $$

for every fixed $r \in\varOmega$ with

31 $$ \psi_{r}(t) := \psi(r,t), \quad t \in[0,T], $$

and

32 $$ w_{r} := w\bigl(r,r'\bigr), \quad r,r' \in \varOmega. $$

Equation (30) is a *family of integral equations* for the unknown kernel $w(r,r')$, where each function $w_{r} = w(r,\cdot)$ provides a different integral equation with a different integral kernel and a different left-hand side. Its structure is given by the integral operator

33 $$ (V_{r}g) (t) : = \int_{\varOmega}K_{r}\bigl(t,r'\bigr) g \bigl(r'\bigr) \,dr', \quad t \in[0,T], $$

with kernel

34 $$ K_{r}\bigl(t,r'\bigr) := \phi \bigl(r',t-D\bigl(r,r'\bigr)\bigr), \quad \bigl(t,r'\bigr)\in[0,T]\times\varOmega, $$

for $r \in\varOmega$. For $N>1$ this kernel is a vector of functions $\phi^{(\xi)}(r',t-D(r,r'))$ with $\xi=1,\ldots,N$. Now, our inverse problem equation (30) is given by

35 $$ \psi_{r} = V_{r} w_{r} $$

for $r \in\varOmega$. For each $r\in\varOmega$ equation (35) is a Fredholm integral equation of the first kind with continuous kernel *ϕ*. The operator $V_{r}$ is a compact operator on the spaces $C(\varOmega)$, $L^{1}(\varOmega)$ or $L^{2}(\varOmega)$ into $\operatorname{BC}([0,T])$. It is well known (cf. [21, 25, 27, 37]) that this equation is *ill posed*, i.e. it does not need to have unique solutions and if it has a solution in general this solution does not depend continuously on the right-hand side.

Ill-posed equations need some regularization method (cf. [26]) in order to obtain a stable solution. A standard approach to regularization is built on the singular system (cf. [27]) of the operator under consideration. In summary, for a compact linear operator $A:X \to Y$ between Hilbert spaces *X* and *Y*, and its adjoint $A^{*}$, the singular values $\mu_{n}$ of the operator *A* are the non-negative square roots of the eigenvalues of the self-adjoint compact operator $A^{*}A: X \to X$. This leads to a representation of the operator as a multiplication of two orthonormal systems ${g_{n} :n \in\mathbb{N}}$ in *X* and ${y_{n} : n \in\mathbb{N}}$ in *Y*. Hence, this corresponds to a spectral representation of the operator *A* in the form

36 $$ Ag=\sum_{n=1}^{\infty}{\mu_{n} \langle g,g_{n} \rangle y_{n}}, $$

for $g \in X$. For the orthonormal systems $g_{n}$ and $y_{n}$ we obtain

37 $$ Ag_{n}=\mu_{n} y_{n}, \qquad A^{*}y_{n}= \mu_{n} g_{n} . $$

Here, in the case *A* that is injective, the inverse of *A* is given by

38 $$ A^{-1}y=\sum_{n=1}^{\infty}{ \frac{1}{\mu_{n}} \langle y,y_{n} \rangle g_{n}} $$

or, if *A* is not injective, the inverse $A^{-1}$ in equation (38) projects onto the orthogonal space $N(A)^{\perp}= \{ g| \langle g,g^{*} \rangle=0, \forall g^{*} \in N(A) \}$. Because of the compactness of the operators *A*, the singular values are a sequence mostly accumulating at zero. So, the behaviour of $|\frac{1}{\mu_{n}} | \rightarrow\infty$, $n \rightarrow \infty$ enlarges small errors causing the instability of applying the inverse. The practical behaviour of the sequence of singular values $\mu_{n}$ provides important insight into the nature of the instability. For the application at hand the problem is strongly ill posed for strong smoothness of the function *ϕ*.

To deal with this instability, we apply regularization techniques to minimize the value of the factor $\frac{1}{\mu_{n}}$ for large *n*. We replace it by another factor $q_{n}$ which is bounded for $n \in\mathbb{N}$, and we modify the inverse operator by

39 $$ R_{\alpha}y = \sum_{n=1}^{\infty}q_{n}^{(\alpha)}\langle y,y_{n} \rangle g_{n}, $$

where $\alpha>0$ is known as regularization parameter and the specific choice of damping factors

40 $$ q_{n}^{(\alpha)} := \frac{\mu_{n}}{\alpha+ \mu_{n}^{2}}, \quad n \in \mathbb {N}$$

leads to the famous Tikhonov regularization (see for example [21, 25–27]).

#### Theorem 4.1

*Let* $u(r,t)$ *for* $r\in\varOmega$ *and* $t\in[0,T]$ *be some neural activity function*, *which obeys the neural field equation* (3) *with true kernel* $w^{\ast}$ *and some initial conditions* $u(r,t)=u_{0}(r,t)$ *for* $(r,t) \in\varOmega\times[-c_{T},0]$. *Then the application of the Tikhonov regularization* (39) *to the integral equation* (35) *leads to the reconstruction* $w_{\alpha}(r,r')$ *of* $Pw^{\ast}$, *where* *P* *is applied to the second argument of* $w(r,r')$ *as the projection of* $w^{\ast}_{r}$ *onto* $N(V_{r})^{\perp}$, *i*.*e*., *it is defined as*

41 $$ (Pw) (r,\cdot) = P_{r}w_{r}, \quad r \in\varOmega. $$

#### Proof

Here, we base our reconstruction on a well-known result (cf. [21], Theorem 3.1.8) that states that Tikhonov regularization is a regularization scheme in the sense of Definition 3.1.4 of [21], i.e., that if $f = A(\varphi^{\ast}) \in R(A)$, then $R_{\alpha}f \rightarrow \varphi^{\ast}$ for $\alpha\rightarrow0$. If *A* is not injective, splitting the space into $N(A)$ and $N(A)^{\perp}=\overline{A^{\ast}(X)}$, we see by $w_{r} = Pw_{r} + (I-P)w_{r}$ and $A^{\ast}$ that the convergence of $R_{\alpha}f$ is towards the projection $P\varphi^{\ast}$ of $\varphi^{\ast}$ onto $N(A)^{\perp}$. In our case, the reconstruction calculates an approximation to $Pw^{\ast}_{r}$. This completes the proof. □

Usually, Tikhonov regularization is carried out by applying an efficient solver^1^ to the equation

42 $$ \bigl(\alpha I + A^{\ast}A\bigr) g = A^{\ast} y, $$

which is equivalent to the spectral version of equation (39). Equation (42) is used for our numerical examples of the subsequent section.

### Sensitivity Analysis

An important basic question is the influence of noise on the reconstruction. Here, we carry out a *sensitivity analysis*, i.e. we calculate the Fréchet derivative of the reconstructed kernel with respect to the input function *u*. Differentiability is obtained in a straightforward manner following [21], Chap. 2.6.

We start with equation (35), where the operator $V_{r}$ and the right-hand side $\psi_{r}$ depend on the input function *u*. The reconstruction of *w* is carried out by the regularized version of

43 $$ w_{r} = (V_{r})^{-1} \psi_{r}, $$

which in the case of Tikhonov regularization (42) is

44 $$\begin{aligned} \begin{aligned}[b] w_{r,\alpha} & = R_{\alpha} \psi_{r} \\ & = \bigl(\alpha I + V_{r}^{\ast} V_{r} \bigr)^{-1} V_{r}^{\ast} \psi_{r}. \end{aligned} \end{aligned}$$

We differentiate with respect to *u* on both sides and employ the chain rule and Eq. (2.6.21) of [21], to derive the *unregularized* form

45 $$ \frac{\partial w_{r}}{\partial u} = - (V_{r})^{-1} \frac{\partial V_{r}}{\partial u} (V_{r})^{-1} \psi_{r} + (V_{r})^{-1} \frac{\partial\psi_{r}}{\partial u} $$

and the derivative of the *regularized* reconstruction

46 $$\begin{aligned} \frac{\partial w_{r,\alpha}}{\partial u} = & - Q \frac{\partial (V_{r}^{\ast} V_{r})}{\partial u} Q V_{r}^{\ast} \psi_{r} + Q \frac{\partial V_{r}^{\ast}}{\partial u} \psi_{r} + Q V_{r}^{\ast} \frac{\partial\psi_{r}}{\partial u} \\ = & - Q \frac{\partial V_{r}^{\ast}}{\partial u} V_{r} Q V_{r}^{\ast} \psi_{r} - Q V_{r}^{\ast} \frac{\partial V_{r} }{\partial u} Q V_{r}^{\ast} \psi_{r} \\ &{}+ Q \frac{\partial V_{r}^{\ast}}{\partial u} \psi_{r} + Q V_{r}^{\ast} \frac{\partial\psi_{r}}{\partial u}, \end{aligned}$$

where we use the notation

47 $$ Q_{r} := \bigl(\alpha I + V_{r}^{\ast} V_{r}\bigr)^{-1}. $$

The derivatives of $V_{r}$ and $\psi_{r}$ with respect to *u* are calculated as follows, where we restrict our presentation to the case where we are given *one* trajectory only. The operator $V_{r}$ in its dependence on *u* is given by

48 $$ \bigl(V_{r} [u] g\bigr) (t) = \int_{\varOmega} f\bigl[ u\bigl(r',t-D \bigl(r,r'\bigr)\bigr) \bigr] g\bigl(r'\bigr) \,dr', \quad t \in[0,T], $$

leading to the Fréchet derivative

49 $$\begin{aligned} &\biggl(\frac{\partial V_{r}[u]}{\partial u}(\delta u) g\biggr) (t) \\ &\quad = \int_{\varOmega} f'\bigl[ u\bigl(r',t-D \bigl(r,r'\bigr)\bigr) \bigr] \delta u\bigl(r',t-D \bigl(r,r'\bigr)\bigr) g\bigl(r'\bigr) \,dr',\quad t \in[0,T], \end{aligned}$$

where $f'$ denotes the derivative of the function $f(s)$ with respect to its real argument $s\in \mathbb {R}$. We need to assume that *f* is differentiable and that the derivative is continuous and bounded. The derivative of the adjoint $V_{r}^{\ast}$ with respect to the $L^{2}$ scalar products on *Ω* and $[0,T]$, which is

50 $$ \bigl(V_{r}^{\ast} [u] \eta\bigr) \bigl(r'\bigr) = \int_{0}^{T} f\bigl[ u\bigl(r',t-D \bigl(r,r'\bigr)\bigr) \bigr] \eta(t) \,dt, \quad r' \in \varOmega, $$

is given by

51 $$ \biggl(\frac{\partial V_{r}^{\ast}[u]}{\partial u}(\delta u) \eta \biggr) \bigl(r'\bigr) = \int_{0}^{T} f'\bigl[ u \bigl(r',t-D\bigl(r,r'\bigr)\bigr) \bigr] \delta u \bigl(r',t-D\bigl(r,r'\bigr)\bigr) \eta(t) \,dt, $$

for $r'\in\varOmega$. We note that $V_{r}^{\ast}$ is an operator into $L^{1}(\varOmega)$, which depends bounded continuously on $r\in\varOmega$. The Fréchet derivative of the function $\psi_{r}$ given by (26) is readily seen to be given by

52 $$ \frac{\partial\psi_{r}}{\partial u}(\delta u) = \tau\frac{\partial \delta u}{\partial t}(r,t) + \delta u(r,t), $$

for $(r,t) \in\varOmega\times[0,T]$. We summarize the results in the following theorem.

#### Theorem 4.2

*Assume that the activation function* *f* *is continuously differentiable with derivative* $f'$ *bounded on* $\mathbb {R}$. *Then*, *for each fixed* $\alpha>0$, *the regularized reconstruction of the kernel* *w* *from input signals* *u* *within the framework of the delay neural field equation is continuously Fréchet differentiable with respect to* *u* *considered as mapping from* $\operatorname{BC}(\varOmega)\times C^{1}([0,T])$ *into* $\operatorname {BC}(\varOmega)\times L^{1}(\varOmega)$. *This implies continuity of the mapping of* *u* *onto* *w*. *The total derivative of* $w_{r}$ *with respect to* *u* *is obtained by the combination of* (46) *with* (49), (51) *and* (52).

#### Proof

Differentiability follows from the differentiability of all the operators in (46) following equations (46) to (52) of the above arguments. □

## Numerical Examples

The goal of this section is to demonstrate the feasibility of the inverse method for the reconstruction of spatial kernels based on the spatio-temporal neural field activity. We study the feasibility in Sect. 5.1 and the sensitivity with respect to variations in the input function *u* in Sect. 5.2.

### Feasibility of Kernel Reconstructions

First, we consider a one-dimensional manifold embedded in a two-dimensional space, illustrating the method for a case with 10,000 degrees of freedom. Then an example involving a two-dimensional spatial domain evaluates the method for an inverse problem with more than 200,000 degrees of freedom for the kernel estimation.

We first need to consider the role of boundaries in the neural field model equation (3) and its examples. For any distribution of neurons in space some activity $u(r,t)$ depending on time *t* can be defined. Mutual influence in space is given by the integral in equation (3). In contrast to models based on partial differential equations, there is no direct boundary effect in these equations. However,

- if one uses a local kernel $w(r,r')$ with strong connectivity only in a neighbourhood of *r*, boundary effects for neurons close to the boundary of the domain will appear, since less neurons are included in a neighbourhood there; whereas
- if the activity of neurons close to the boundary is close to zero, usually such boundary effects remain negligible.

We will study a setup which avoids boundary effects by the choice of an embedding of a one-dimensional manifold into two-dimensional space in our first example, where there are always the same number of neurons in a neighbourhood of any neuron on the whole manifold. The second example instead limits boundary effects by using only small excitations close to the boundary in a two-dimensional neural patch.

#### Example 1

**Fig. 1:**
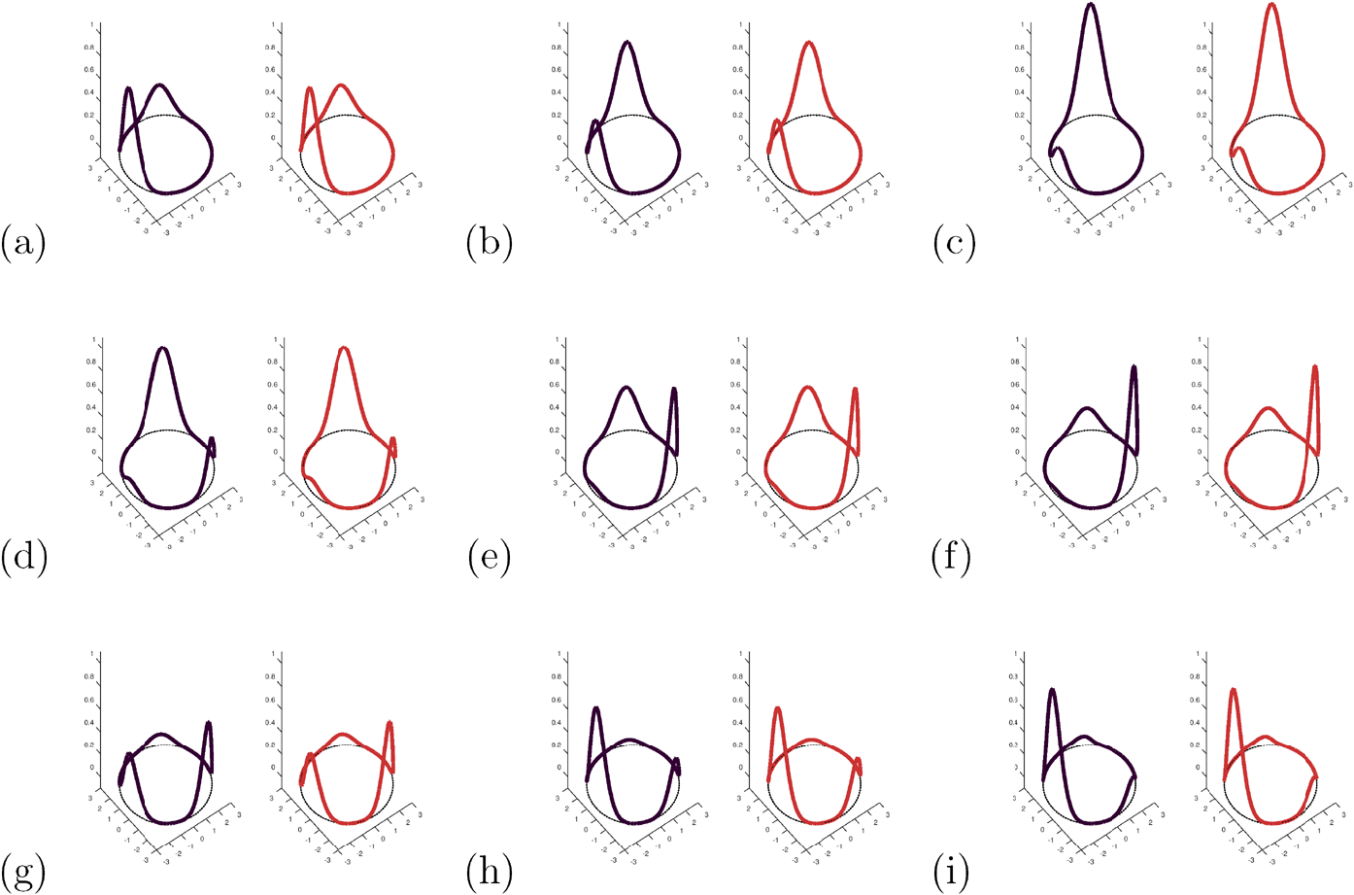
Original and Reconstructed Time Sequence 1D. Time sequence of excitation of the one-dimensional delay neural field. The original field is shown in black, in red the dynamics based on the delay kernel reconstruction. One cycle of the oscillation is shown at time steps 1, 3, 6, 10, 13, 16, 19, 22, 25, with a step size of $\Delta t=0.2$, in panels **(a)** to **(i)**

We start with a simple one-dimensional closed curve or manifold, respectively, embedded in a two-dimensional space. In particular, we study the dynamics of the activity field $u(r,t)$ on the boundary $\partial B_{R} \subset\mathbb{R}^{2}$ of a disk with radius *R*, as displayed in Fig. 1. Here we consider that $v=1$, and use a simple and smooth delay function for $r=(x,y)$ and $r'=(x',y')$ with $r,r' \in \partial B_{R}$ based on the embedding into $\mathbb {R}^{2}$ which is defined by

53 $$ D\bigl(r,r'\bigr) := \tilde{D}\bigl(r,r'\bigr) = \bigl\vert r-r' \bigr\vert = \sqrt{ \bigl(x-x' \bigr)^{2} + \bigl(y-y'\bigr)^{2}}, \quad r,r' \in\partial B_{R}. $$

This simple sandbox for testing our method hence can be considered as neurons growing on the boundary of a disk, but connecting directly through its interior. This is reminiscent of the thin exterior layer of grey matter containing neurons connecting through an interior bulk of white matter containing axons in the brain. However, we point out that this is a different setup from previous studies that superficially appear similar, where the spatial domain instead is a ring with periodic boundary conditions [38, 39].

We implemented the delay neural field equation in MATLAB^®^ based on an Euler method for the time-evolution of the system with zeroth-order or first-order quadrature (rectangular rule or trapezoidal rule) for the integral parts of the integro-differential equation. For the purposes of studying the kernel reconstruction on a rather short temporal window this simple approach is completely sufficient and does not show any deficiencies compared to higher-order methods for the forward problem, as employed for example in [21, 28, 40].

We first solve the direct problem, i.e., calculate the time-evolution of the excitation field $u(r,t)$. As initial condition, we choose the exponential function

54 $$ u(r,0) = e^{-\sigma|r-r_{0}|^{2}},\quad r \in\partial B_{R}. $$

**Fig. 2:**
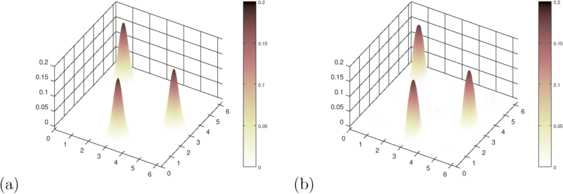
Reconstructed Kernel 1D-Case. For the one-dimensional example the kernel can be visualized as a two-dimensional scalar function $w(r,r')$. We display **(a)** the original and **(b)** the reconstructed kernel of the one-dimensional delay dynamics shown in Fig. 1

We prescribe a neural kernel of the form

55 $$\begin{aligned} w\bigl(r,r'\bigr) =& c \bigl( e^{-\tau|r-r_{1}|^{2}} e^{-\tau|r'-r_{0}|^{2}} + e^{-\tau|r-r_{2}|^{2}} e^{-\tau|r'-r_{1}|^{2}} \\ &{} + e^{-\tau|r-r_{0}|^{2}} e^{-\tau|r'-r_{2}|^{2}} \bigr) \end{aligned}$$

for $r,r' \in\partial B_{R} \subset\mathbb{R}^{2}$ with constants $c>0$ and $\tau>0$. The full set of values used for our simulations are given in Table 1. This leads to delayed excitation of areas around three points $r_{0}, r_{1}$ and $r_{2}$ equally distributed on a circle, where, with some delay, the excitation field around $r_{0}$ will excite the field around $r_{1}$, the field around $r_{1}$ will excite the field around $r_{2}$ and the field around $r_{2}$ will excite the field around $r_{0}$ again. The function *f* is chosen to be sigmoidal as in equation (6). We have generated a classical oscillator, as can be seen in the snapshots in Fig. 1 (black curves). Its kernel is visualized in Fig. 2(a).

**Table 1:** Parameter values for Example 1. Simulations have been carried out with $N=101$ equally distributed nodes on the circle, $N_{t}=50$ time steps, and a time step size $\Delta t=0.2$ for the inverse problem

$r_{0}$	(cos(*π*),sin(*π*))	*σ*	1.0
$r_{1}$	(cos(*π*/3),sin(*π*/3))	*τ*	1.0
$r_{2}$	(cos(−*π*/3),sin(−*π*/3))	*c*	3.0

Next we reconstruct the kernel by the inverse problem technique from the so obtained temporal evolution of the excitation field $u(r,t)$ for some time window $t \in[0,T]$ according to equations (30) and (39). Given a discretized version of $u(r,t)$ on nodes

56 $$ r_{\ell} := \biggl(\cos\biggl(\frac{2\pi\cdot\ell}{N} \biggr), \sin\biggl( \frac{2\pi\cdot\ell}{N} \biggr) \biggr),\qquad t_{k}=k\cdot \Delta t, $$

for $\ell=0, \ldots, N$ and $k=0, \ldots, N_{t}-1$, we calculate *ϕ* and *ψ* according to equations (25) and (26) and then employ the regularization (39) via (42) to solve equation (35) for $r \in\partial B_{R}$. In Fig. 2, we compare the original with the reconstructed kernel in the case where no additional noise is added, carried out with $\alpha=0.01$ and find a very good agreement between both.

As a test, we employ the reconstructed kernel with the same initial condition to calculate a reconstructed neural field $u_{\mathrm{rec}}(r,t)$ on $(r,t)\in\partial B_{R}\times[0,T]$. The original dynamics is shown in black in Fig. 1, based on the kernel (55) visualized in Fig. 2(a). The reconstructed dynamics is shown in red in Fig. 1, based on the reconstructed kernel visualized in Fig. 2(b). A very good agreement between original and reconstructed solution is observed.

#### Example 2

**Fig. 3:**
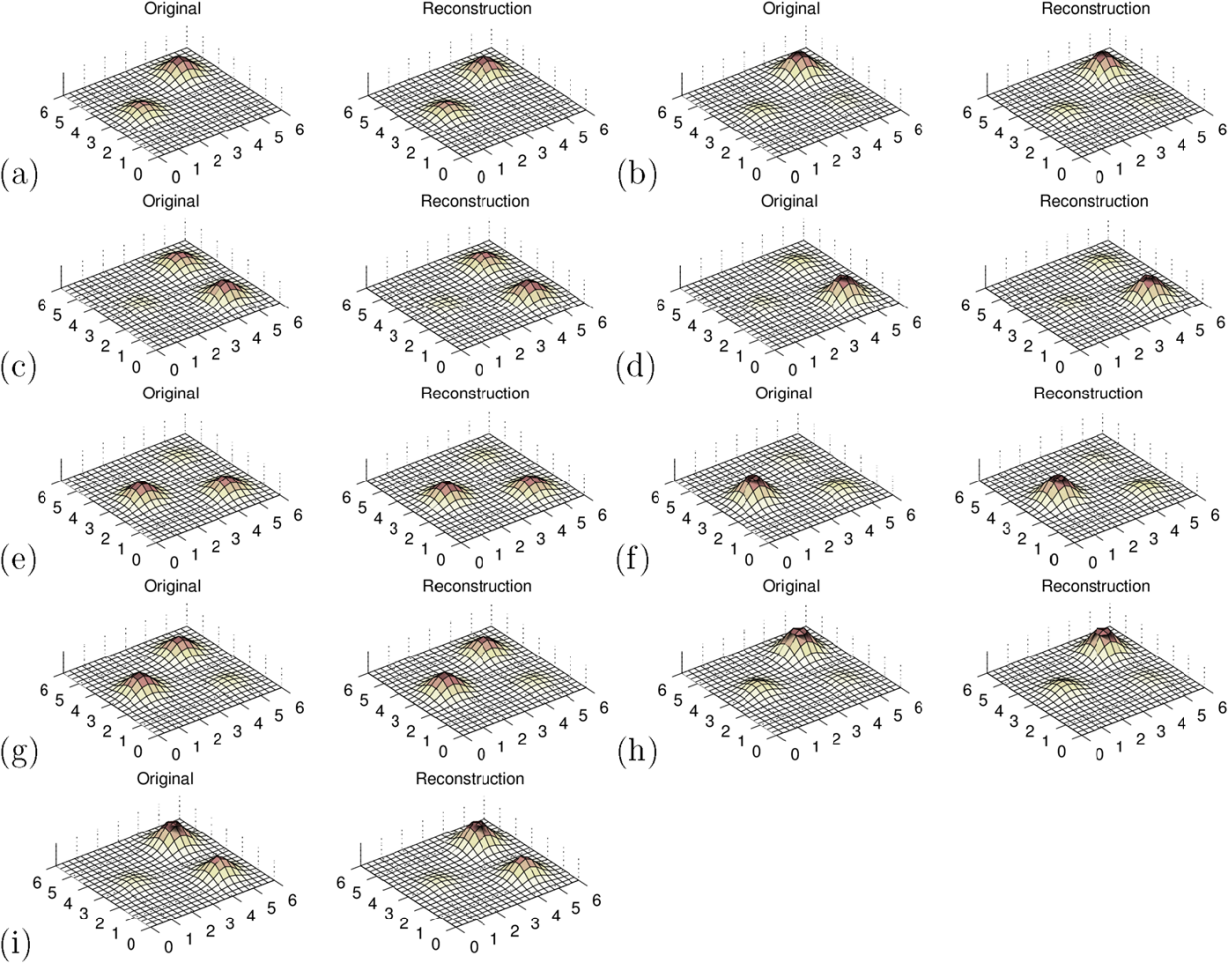
Original and Reconstructed Time Sequence 2D. Selection of time slices for the two-dimensional delay neural field. We display time steps 3, 6, 9, 12, 15, 18, 21, 24, 27 with $\Delta t=0.2$ to show one and a half cycles of the oscillation in panels **(a)** to **(i)**. Each panel shows the original on the *left* and simulation with the reconstructed kernel on the *right*

**Fig. 4:**
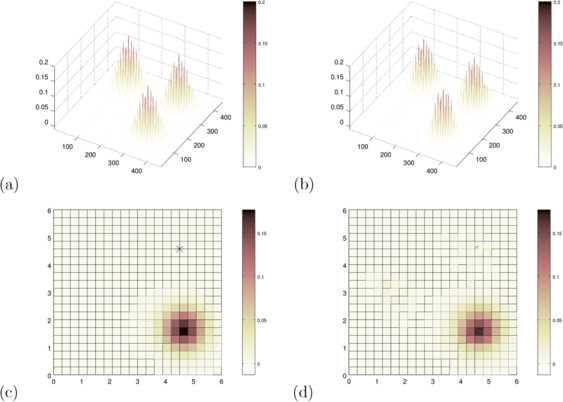
Original and Reconstructed Kernel 2D-Case. We display **(a)** the original and **(b)** the reconstructed kernel of the two-dimensional neural delay dynamics shown in Fig. 3. The images **(c)** and **(d)** show a column of the original and reconstructed kernel, visualizing the connection from the point indicated by the *black star* to the rest of the neural patch

As a second example, we study oscillating two-dimensional neural field activity. Here, the dimension of the state space is higher with $N = 21 \times22 = 462$ spatial elements as shown in Figs. 3 and 4. Our approach is analogous to the one-dimensional example, but now with 213,444 degrees of freedom for the possible connectivity values (see Table 2). We first simulate the neural field dynamics based on equation (3) on a neural patch described by $\varOmega:= [a_{1}, b_{1}]\times[a_{2}, b_{2}] = [0,6]\times [0,6]$. Time slices of this dynamical evolution are displayed in Fig. 3. The kernel has been chosen to be of a form similar to equation (55), but now with points $r_{0}$, $r_{1}$ and $r_{2}$ in the two-dimensional neural patch. This leads to an oscillating field in an area around these points $r_{j}$ with $j=0,1,2$. The activation function *f* is chosen to be sigmoidal again. The initial condition is a Gaussian excitation around the point $r_{0}$. For our simple tests, we again employ zeroth or first-order quadrature and Euler’s method to carry out the simulation.

**Table 2:** Parameter values for Example 2. Simulations have been carried out with $N=21\times22=462$ nodes, $N_{t}=30$ time steps with time step size $\Delta t=0.2$ for the inverse problem. The kernel estimation problem has 213,444 degrees of freedom

$r_{0}$	(1.5,3.0)	*σ*	2.0
$r_{1}$	(4.5,4.5)	*τ*	1.0
$r_{2}$	(4.5,1.5)	*c*	2.1

The kernel $w(r,r')$ with $r,r' \in\varOmega$ now lives on a subset $U:= \varOmega\times\varOmega$ of a four-dimensional space, since *Ω* is a subset of a two-dimensional patch. Visualization of $w(r,r')$ can be carried out by either fixing $r'$ and showing a two-dimensional surface plot, or by re-ordering *r* and $r'$ into one-dimensional vectors, so that $w(r,r')$ can be displayed in full as a two-dimensional surface. The first approach is chosen in Fig. 4(c), where the white star indicates $r'$. The second approach is shown in Fig. 4(a). Next, we solve the *inverse delay neural problem* and reconstruct the kernel based on equation (30) regularized as indicated by equations (39) and (42). Again, this is carried out by calculation of *ϕ* and *ψ* first according to equations (25) and (26), then solving equation (35) by regularization via equation (39) with the regularization parameter chosen as $\alpha=0.1$. This choice leads to a reasonable stability of the reconstructions combined with high reconstruction quality, and it has been chosen by trial and error.

Figures 4(c) and 4(d) display the *original* and the *reconstructed* kernel column, which represents the impact of the location at the black star to all other spatial locations of the neural patch. The result as displayed in (d) shows that the regularized reconstruction of the delay neural kernel is not perfect. However, it is working well if the field activity reaches specific parts of the neural environment. Otherwise the reconstruction is just zero due to missing input for the reconstruction equations and the regularization chosen here. The regularization penalizes the distance to the zero kernel function. Therefore, the results clearly demonstrate the feasibility of the method.

### Sensitivity with Respect to Functional Input

In this section we will carry out a numerical sensitivity study of our first example to explore the dependence of the kernel reconstructions on the input function *u*. It complements our sensitivity analysis of Sect. 4.2.

**Fig. 5:**
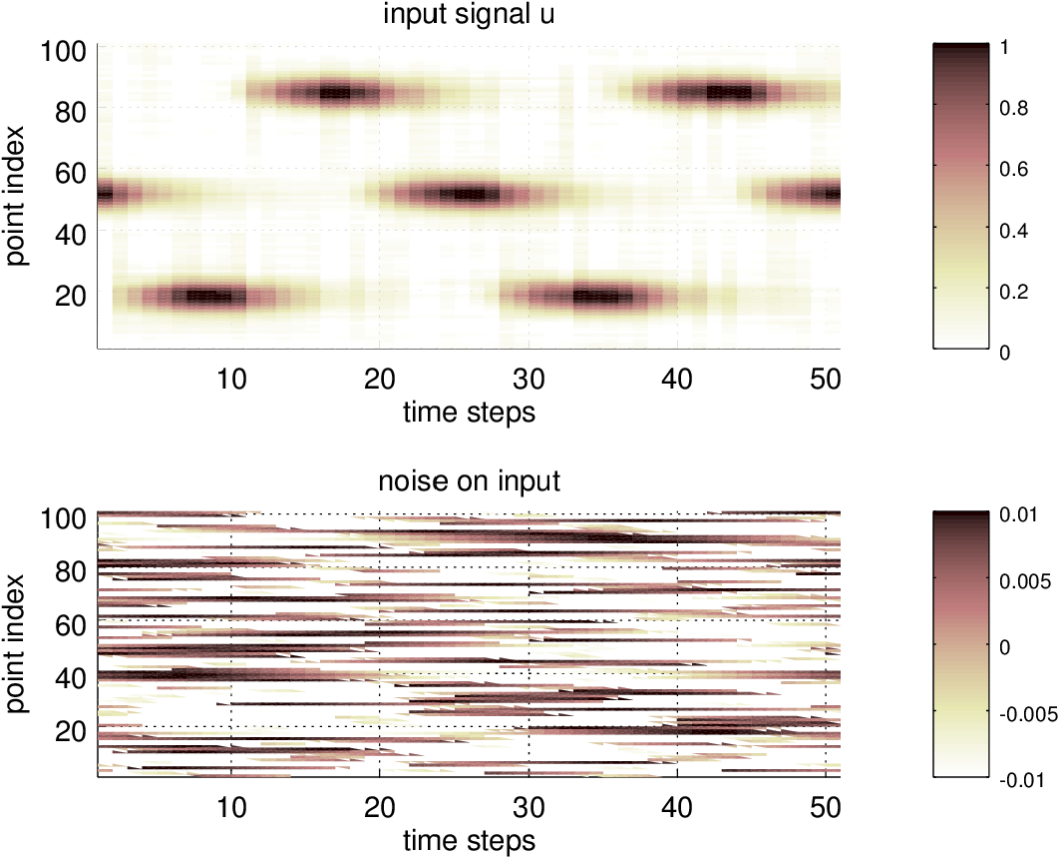
Measured Signal and Measurement Error. In the upper image we display the input signal $u(r,t)$ independence of the point index of the discretized vector *r* and the temporal evolution $t\in[0,T]$. The lower image shows the measurement error which has been added to the signal before a reconstruction has been carried out

We study the stability of the reconstruction when we add some random error to the measured signal $u(r,t)$ displayed in Fig. 1. We remark that we need measurements of our signal which are differentiable with respect to time, since the calculation of *ψ* in (26) includes the temporal derivative of the signal. In practical situations, this would be achieved by a temporal smoothing of the signal. Here, for testing the sensitivity we have added a random shift of a temporally smooth signal in each of the analysis points. The amplitude of the signal is given by $\varepsilon =0.01$, which corresponds to noise of 1% added to the measured temporal signal; compare Fig. 5.

**Fig. 6:**
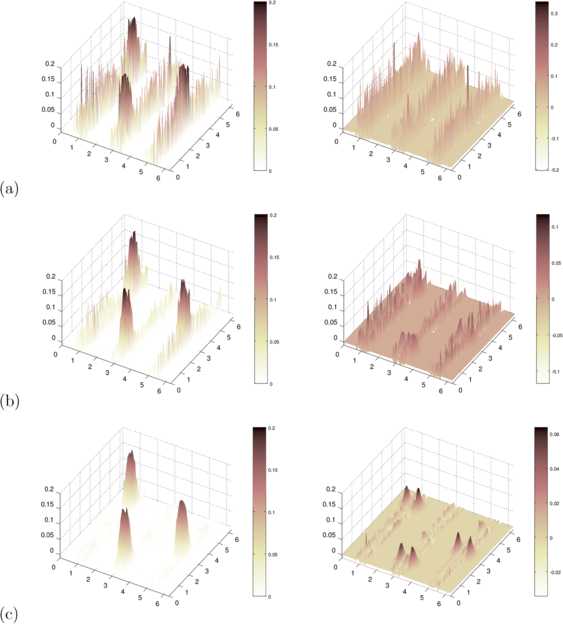
Sensitivity Study of the Influence of Measurement Error. We show reconstruction kernels and the reconstruction error for 1% noise shown in Fig. 5 with regularization parameters $\alpha=0.01$ in **(a)**, $\alpha=0.1$ in **(b)** and $\alpha=1$ in **(c)**. A sufficient reconstruction quality is achieved with $\alpha=1$

Now, we study reconstructions with different regularization parameters *α*, where larger *α* means we regularize in a stronger way, damping the error which comes from the measurement error. Figure 6 displays three different choices of *α*, where $\alpha=1$ leads to reasonable reconstructions, $\alpha=0.1$ shows kernel reconstruction still disturbed by noise, and $\alpha=0.01$ does not lead to satisfactory reconstructions at all.

According to Theorem 4.2 we have continuity of $u\mapsto w$, such that if we lower the error *ε* for fixed *α*, we need to have convergence to the reconstructed kernel in the case of no data error. Indeed, we obtain a figure similar to Fig. 6 when we lower the error parameter *ε* from $\varepsilon =0.01$ to $\varepsilon =0.005$ and $\varepsilon =0.001$, leading to the reconstruction displayed in Fig. 2(b) for $\varepsilon =0$.

## Conclusions

The purpose of this work is to develop an *integral equation approach* for kernel reconstructions in delay neural field equations and to study its practical feasibility. We simulate the activity and evolution of a delayed neural field of Amari-type to develop an effective approach to reconstructing the neural connectivity. As a preparation for the inverse problem, this work includes an explicit study of the solvability of the direct problem of the delayed neural field equation (3). We provide an easily accessible functional analytic approach based on an integral equation and Banach’s fixed-point theorem.

As our main result, we apply inverse problems techniques to reconstructing the neural kernel assuming that some measurements of the activity $u(r,t)$ are given. We start by formulating a *family of integral equations* of the *first kind*. Since kernel reconstruction is ill posed, we need regularization to obtain stable solutions. As stabilization method we employ the Tikhonov regularization. A *sensitivity analysis* is carried out, showing that the mapping of the input *u* to the regularized kernel reconstruction is Fréchet differentiable. The derivative is explicitly calculated based on the integral equation approach.

Finally, we provide numerical examples in one- and two-dimensional spatial domains. These examples show that the regularized reconstruction of the delay neural kernel is practically feasible. We study the numerical sensitivity, by adding random noise of size *ε* (testing 1%, 0.1% and 0.01%) and studying the regularized reconstruction with different regularization parameters.

In this work, we assume the delay function *D* to be given, as it would be the case when the delay is approximately proportional to the distance of the nodes under consideration. If *D* is unknown, *w* is known and *u* is measured, we can solve in equation (3) for $u(r',t-D(r,r'))$ for all *r*, $r'$ and *t*. This is still ill posed, since it involves an integral equation of the first kind, but then the determination of *D* is reduced to the reconstruction of *D* from the knowledge of $u(r',t-D(r,r'))$, which strongly depends on the form of the signal *u* and conditions we impose on *D*. If neither the delay *D* nor *w* would be given, the kernel $K_{r}$ of operator $V_{r}$ would be unknown and part of the reconstruction, leading to many open questions of feasibility and observability. In general, the reconstruction of both the kernel *w* and the delay *D* is an important nonlinear, far reaching and challenging problem of future research.

In summary, we have developed a *stable* and *efficient* approach for the reconstruction of the *connectivity* in neural systems based on *delay neural field equations*. We expect the approach to be extensible to a wide range of field models with delay, and in particular to be highly useful for analyses of experimental data in the domain of computational neuroscience. These methods allow for the reconstruction of the underlying ‘synaptic footprint’ of connectivity from available neural activity measurements, thus providing a basis for simulation and prediction of real phenomena in the neurosciences.

## Footnotes

1 For large-scale problems a conjugate-gradient method is used for solving the equation sequentially. For smaller problems matrix inversion by Gauss’ method is sufficient.

## References

[1] Coombes S, beim Graben P, Potthast R, Wright J (2014). Neural fields: theory and applications.

[2] Wilson HR, Cowan JD (1972). Excitatory and inhibitatory interactions in localized populations of model neurons. Biophys J.

[3] Wilson HR, Cowan JD (1973). A mathematical theory of the functional dynamics of cortical and thelamic nervous tissue. Kybernetik.

[4] Amari S (1977). Dynamics of patterns formation in lateral-inhibition type neural fields. Biol Cybern.

[5] Boeree CG. The neurons. General Psychology. 2015. p. 1–6.

[6] Bressloff PC, Coombes S (1997). Physics of the extended neuron. Int J Mod Phys B.

[7] Nogaret A, Meliza CD, Margoliash D, Abarbanel HDI (2016). Automatic construction of predictive neuron models through large scale assimilation of electrophysiological data. Sci Rep.

[8] Gils S, Janssens SG, Kuznetsov Y, Visser S (2013). On local bifurcations in neural field models with transmission delays. J Math Biol.

[9] Venkov NA. Dynamics of neural field models [PhD thesis]. 2008.

[10] Faye G, Faugeras O (2010). Some theoretical and numerical results for delayed neural field equations. Physica D.

[11] Atay FM, Hutt A (2005). Stability and bifurcations in neural fields with finite propagation speed and general connectivity. SIAM J Appl Math.

[12] Potthast R, beim Graben P (2010). Existence and properties of solutions for neural field equations. Math Methods Appl Sci.

[13] Venkov NA, Coombes S, Matthews PC (2007). Dynamic instabilities in scalar neural field equations with space-dependent delays. Physica D.

[14] Veltz R, Faugeras O (2011). Stability of the stationary solutions of neural field equations with propagation delays. J Math Neurosci.

[15] Veltz R, Faugeras O (2013). A center manifold result for delayed neural fields equations. SIAM J Math Anal.

[16] beim Graben P, Potthast R (2009). Inverse problems in dynamic cognitive modeling. Chaos, Interdiscip J Nonlinear Sci.

[17] Freitag MA, Potthast RWE (2013). Synergy of inverse problems and data assimilation techniques. Large scale inverse problems.

[18] Potthast R. Inverse problems and data assimilation for brain equations—state and current challenges. 2015.

[19] Potthast R (2013). Inverse problems in neural population models. Encyclopedia of computational neuroscience.

[20] Potthast R, beim Graben P (2009). Dimensional reduction for the inverse problem of neural field theory. Front Comput Neurosci.

[21] Nakamura G, Potthast R (2015). Inverse modeling: an introduction to the theory and methods of inverse problems and data assimilation.

[22] Hutt A (2007). Generalization of the reaction-diffusion, Swift-Hohenberg, and Kuramoto-Sivashinsky equations and effects of finite propagation speeds. Phys Rev E.

[23] Coombes S, Venkov N, Shiau L, Bojak I, Liley D, Laing C (2007). Modeling elactrocortical activity through improved local approximations of integral neural field equations. Phys Rev E.

[24] Dijkstra K, van Gils SA, Janssens SG (2015). Pitchfork-Hopf bifurcations in 1D neural field models with transmission delays. Physica D.

[25] Engl HW, Hankle M, Neubauer A (2000). Regularization of inverse problems.

[26] Groetsch CW (1993). Inverse problems in the mathematical sciences.

[27] Kress R (1999). Linear integral equations.

[28] Coombes S, beim Graben P, Potthast R (2014). Tutorial on neural field theory. Neural fields: theory and applications.

[29] James MP, Coombes S, Bressloff PC. Effects of quasioctive membrane on multiply periodic travelling waves in integrate-and-fire systems. 2003. 10.1103/PhysRevE.67.051905 12786176

[30] Laing CR, Coombes S. The importance of different timings of excitatory and inhibitory pathways in neural field models. 2005. 10.1080/09548980500533461 16818395

[31] Bojak I, Liley DT (2010). Axonal velocity distributions in neural field equations. PLoS Comput Biol.

[32] Coombes S, Schmidt H. Neural fields with sigmoidal firing rates: approximate solutions. Nottingham e Prints. 2010.

[33] Rankin J, Avitabil D, Baladron J, Faye G, Lloyd DJ. Continuation of localised coherent structures in nonlocal neural field equations. 2013. arXiv:1304.7206.

[34] Bressloff PC, Kilpatrick ZP (2011). Two-dimensional bumps in piecewise smooth neural fields with synaptic depression. SIAM J Appl Math.

[35] Diekmann O (1995). Delay equations: functional-, complex-, and nonlinear analysis.

[36] Hutt A, Buhry L (2014). Study of gabaergic extra-synaptic tonic inhibition in single neurons and neural populations by traversing neural scales: application to propofol-induced anaesthesia. J Comput Neurosci.

[37] Kirsch A (2011). An introduction to the mathematical theory of inverse problems.

[38] Hutt A, Bestehorn M, Wennekers T (2003). Pattern formation in intracortical neural fields. Netw Comput Neural Syst.

[39] Wennekers T (2001). Orientation tuning properties of simple cells in area V1 derived from an approximate analysis of nonlinear neural field models. Neural Comput.

[40] Potthast R, Graben P (2009). Inverse problems in neural field theory. SIAM J Appl Dyn Syst.

